# Calcium carbonate alters the functional response of coastal sediments to eutrophication-induced acidification

**DOI:** 10.1038/s41598-019-48549-8

**Published:** 2019-08-19

**Authors:** Tarn P. Drylie, Hazel R. Needham, Andrew M. Lohrer, Adam Hartland, Conrad A. Pilditch

**Affiliations:** 1 School of Science, University of Waikato, Private Bag 3240, Hamilton, New Zealand; 2 National Institute of Water and Atmospheric Research (NIWA), PO Box 11115, Hamilton, New Zealand

**Keywords:** Biodiversity, Ecosystem ecology, Ecosystem services, Carbon cycle, Marine biology

## Abstract

Coastal ocean acidification research is dominated by laboratory-based studies that cannot necessarily predict real-world ecosystem response given its complexity. We enriched coastal sediments with increasing quantities of organic matter in the field to identify the effects of eutrophication-induced acidification on benthic structure and function, and assess whether biogenic calcium carbonate (CaCO_3_) would alter the response. Along the eutrophication gradient we observed declines in macrofauna biodiversity and impaired benthic net primary productivity and sediment nutrient cycling. CaCO_3_ addition did not alter the macrofauna community response, but significantly dampened negative effects on function (e.g. net autotrophy occurred at higher levels of organic matter enrichment in +CaCO_3_ treatments than −CaCO_3_ (1400 vs 950 g dw m^−2^)). By identifying the links between eutrophication, sediment biogeochemistry and benthic ecosystem structure and function *in situ*, our study represents a crucial step forward in understanding the ecological effects of coastal acidification and the role of biogenic CaCO_3_ in moderating responses.

## Introduction

Acidification of seawater via the increasing absorption of atmospheric CO_2_ (ocean acidification; OA) is a key contemporary issue for the marine environment^1^. However, eutrophication-induced acidification^2^ has received comparatively little attention, despite affecting vast portions of the world’s coastal zones^3^. Eutrophication is defined as an increase in the rate of supply of organic carbon to an ecosystem and occurs in coastal waters primarily via the anthropogenic input of excess nutrients^4^. Excess nutrients promote short-lived algal blooms which, upon collapsing, are deposited to the benthos^5^ where microbially-driven aerobic respiration of organic matter releases CO_2_ in approximate equivalence to O_2_ consumption^6^, causing localised acidification^7^. In highly productive estuarine environments, eutrophication-induced acidification adds variation to background fluctuations in pH driven by changing rates of respiration and photosynthesis^8,9^ and watershed effects^10^, and occurs against a backdrop of global OA that is set to reduce seawater pH 0.3–0.4 units by 2100^11^. The co-occurrence of these acidification pathways means coastal environments may experience decreases in seawater pH far exceeding those predicted from OA alone^12–14^. Additionally, biogeochemical changes associated with aerobic (i.e., O_2_ depletion) and anaerobic (i.e., dissimilatory N and S reduction) pathways of benthic organic matter degradation mean that acidification rarely acts in isolation, but is rather a single component in a multi-stressor setting also comprising hypoxia and increased concentrations of toxic solutes^15,16^.

Coastal benthic macrofauna communities exhibit predictable responses to organic matter enrichment, such as reduced abundance and diversity at high levels of loading^17^. These responses are usually attributed to the onset of hypoxia^3^, however empirical evidence suggests some organisms respond more strongly to fluctuations in pH than O_2_ (e.g. juvenile clams)^18^. Whilst it is generally accepted that calcifying organisms will fare poorly under acidification due to calcium carbonate (CaCO_3_) under-saturation and increased likelihood of dissolution^19–21^, the consequences for non-calcifying organisms are less certain and vary interspecifically^22^. Understanding the response of benthic communities to decreasing pH is critical, as numerous ecosystem services (e.g. food production and nutrient cycling) are underpinned by functions (e.g. primary production and organic matter mineralisation) that are strongly influenced by resident macrofauna^23,24^. Evidence from laboratory experiments suggests that processes such as denitrification may be disrupted by decreases in seawater pH, owing to a breakdown in the facilitative interactions between macrofauna and denitrifying bacteria^25,26^. Naturally occurring gradients of acidification have been utilised to investigate the effects on benthic communities in the field, for example by monitoring communities with increasing distance from shallow volcanic CO_2_ vents^27–29^. Whilst these studies have provided insights into potential changes in community structure, including decreases in the abundance of calcareous organisms, and even evolutionary adaptations, there are frequently additional environmental variables that co-vary along the gradient (such as temperature and toxic trace elements) making it difficult to attribute the responses to acidification alone^30^.

The difficulty in conducting controlled acidification experiments *in situ* has meant benthic OA research has tended towards laboratory-based studies often using simplified biological communities or individual species. Of the 324 studies published by 2014 on marine organisms’ responses to reduced coastal pH, three quarters were conducted in the laboratory^31^. Whilst laboratory-based studies provide a controlled mechanistic understanding of acidification effects, they cannot predict *in situ* ecological responses given the complexity of natural ecosystems. For example, the ability for mobile organisms to disperse from impacted sediments^32^ is inhibited when entire experimental units (e.g. cores or mesocosms) are acidified, but this is a crucially important behavioural trait providing resilience to benthic communities when considered at landscape or ecosystem scales^33,34^. Recent OA field studies have also highlighted how functional redundancy in benthic communities may provide resilience against acidification which would otherwise be masked in low diversity laboratory studies. Baggini *et al*.^35^ documented a switch in the main grazer of macroalgae from sea urchins to fishes along a gradient of increasing CO_2_ concentration, preventing a phase shift from an ecosystem dominated by encrusting algae to macroalgae. Numerous laboratory studies have previously identified positive responses of marine autotrophs to increased CO_2_ (see^36^), but this may not be observed *in situ* if species replacement maintains grazing pressure.

Furthermore, laboratory experiments frequently neglect the multi-stressor nature of benthic acidification (the usual mechanism for reducing seawater pH is via the bubbling of CO_2_ gas^37,38^, shifting water column pH only), and the natural buffering capacity of the coastal environment may be overlooked or poorly replicated in artificial settings. To make empirical studies more relevant to the real-world, Hewitt *et al*.^39^ advocate conducting field experiments across environmental gradients to address scaling issues between the understanding gleaned from simplified small-scale laboratory experiments and the response of diverse large-scale ecosystems^40,41^.

The complex biogeochemistry of the coastal zone is likely to affect ecological responses to acidification. Coastal ocean sediments contain approximately 50% of oceanic CaCO_3_ deposits, originating in temperate regions mostly from bivalve shells and bryozoan skeletons^42^. CaCO_3_ minerals dissolve in response to increased acidity from CO_2_ hydrolysis, according to equations (i) and (ii).

1 $${{\rm{CO}}}_{2({\rm{g}})}+2{{\rm{H}}}_{2}{{\rm{O}}}_{({\rm{aq}})} < \, \mbox{-} \, > {{{\rm{HCO}}}_{3(\mathrm{aq})}}^{-}+{{\rm{H}}}_{3}{{{\rm{O}}}^{+}}_{({\rm{aq}})}$$

2 $${{\rm{CaCO}}}_{3({\rm{s}})}+{{\rm{H}}}_{3}{{{\rm{O}}}^{+}}_{({\rm{aq}})} < \, \mbox{-} \, > {{{\rm{HCO}}}_{{\rm{3}}({\rm{aq}})}}^{-}+{{\rm{H}}}_{2}{{\rm{O}}}_{({\rm{aq}})}+{{{\rm{Ca}}}^{2+}}_{({\rm{aq}})}$$

When CO_2_ hydrolysis and CaCO_3_ dissolution are coupled in this way, there is no net change in hydronium ion (H_3_O^+^) concentration and thus no change in pH (pH = −log[H_3_O^+^]), although buffering capacity, i.e. the ability to neutralise H_3_O^+^, increases according to the increase in HCO_3_^−^ ^43^. The porewaters of CaCO_3_-rich coastal sediments may therefore possess high buffering capacities^44,45^ which reduce the potential for significant fluctuations in pH. CaCO_3_ has even been suggested as a tool to prevent the negative effects of localised benthic acidification^46^. Whilst Green *et al*.^47^ observed a three-fold increase in bivalve recruitment in cores buffered with crushed shell hash, Greiner *et al*.^48^ observed no such effect in similarly buffered plots, despite significant increases in porewater pH. To date, the only field study investigating the link between benthic acidification, CaCO_3_ buffering and ecosystem function was inconclusive due to heterogeneity in the environment^49^. It is necessary to clarify the nature of these linkages as intensifying coastal acidification may remove the key species that consolidate CaCO_3_ into sediments^50^ and lead to dissolution of existing CaCO_3_ deposits^51^, such that any resilience provided by CaCO_3_ buffering to ecosystem functioning will be lost.

In this study, the ability of biogenic CaCO_3_ to maintain intertidal benthic ecosystem structure and function along a gradient of eutrophication-induced acidification was investigated *in situ*. Organic matter enrichment treatments (0–2250 g dw m^−2^, increasing in 250 g increments) formed the eutrophication gradient, and enriched plots (1.44 m^2^) were allocated one of two CaCO_3_ treatments (with or without the addition of 2000 g m^−2^, i.e. +CaCO_3_ or −CaCO_3_ treatments). After 70 d of enrichment we assessed treatment effects on sediment properties, macrofauna community structure and key ecosystem functions (e.g. net primary production and nutrient processing), which were derived from solute fluxes across the sediment–water boundary measured in light and dark benthic incubation chambers. The presumed mechanism by which CaCO_3_ would increase resilience was through reducing the range of pH variability via buffering of CO_2_ (Eqs 1 and 2). As such, it was hypothesised that sediments with abundant CaCO_3_ would experience less extreme fluctuations in pH than those without when mineralising excess organic matter to CO_2_. If fluctuating pH was the primary eutrophication-induced stressor, the macrofauna community was expected to be less affected by organic matter enrichment when there was excess CaCO_3_, helping to maintain ecosystem function. Conversely, a lack of a positive effect of CaCO_3_ could indicate that additional eutrophication-induced stressors (hypoxia, H_2_S toxicity) were more important regulators.

To test our predictions, one-way PERMANOVA (using the PERMANOVA + package of Primer V7) were performed with CaCO_3_ treatment considered a fixed factor and organic matter treatment a continuous co-variable. This approach allowed assessment of the interactive effects of organic matter and CaCO_3_ treatments on response variables. Some ecosystem functions were variable yet significantly correlated with organic matter treatment, therefore multiple regression (using distance-based linear models (DistLM) in Primer V7) was also performed to determine environmental drivers (e.g. sediment properties, macrofauna community structure) of this variability.

## Results

**Figure 1:**
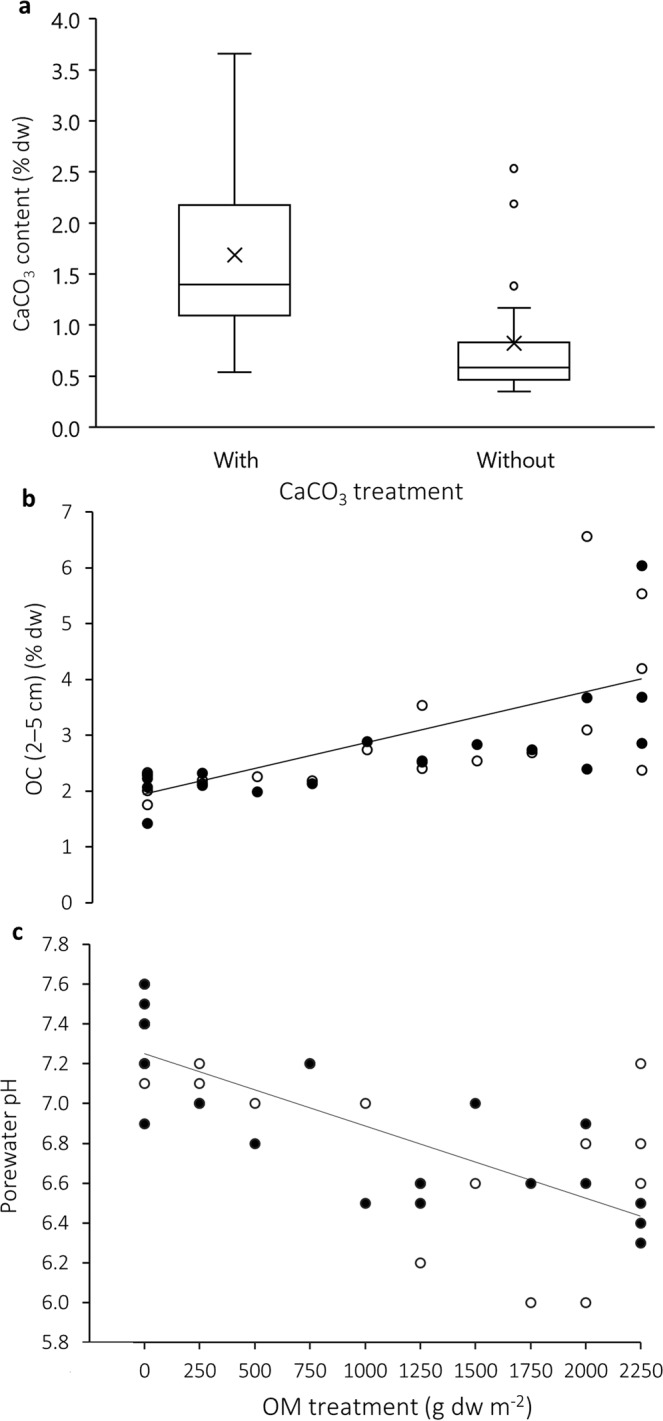
Effects of treatments (CaCO_3_ and organic matter (OM) addition) on sediment (**a**) CaCO_3_ content (t-test, p = 0.001) (**b**) organic content (OC) (r^2^ = 0.44, p < 0.001) and (**c**) porewater pH (r^2^ = 0.47, p < 0.001) 70 d after manipulation. Closed symbols represent −CaCO_3_ treatments and open symbols represent +CaCO_3_ treatments. Regression lines are fitted to the pooled data set (see Table 2). Boxplot whiskers indicate the non-outlier range, with circles marking outliers. Box limits represent 25th and 75th percentiles, lines intersecting boxes are median values and crosses within boxes are means.

Seventy days after manipulation, surface sediment characteristics (0–2 cm) were similar between plots (Table 1) and any effects of the disturbance caused by treatment establishment had subsided (see Supplementary Information for ambient and control treatment comparisons). Only phaeopigments, a product of chlorophyll degradation, showed a relatively large range (2–27 µg g^−1^ dw) driven by high values in organically enriched plots. At 2–5 cm sediment depth (organic matter and CaCO_3_ were added at a depth of 5 cm) a significant elevation of CaCO_3_ content in +CaCO_3_ treatments (1.4%) versus −CaCO_3_ treatments (0.5%) (F(1, 33) = 12.81, p = 0.001; Fig. 1a) was achieved. There was no significant interactive effect between CaCO_3_ and organic matter treatments on organic content (OC) or porewater pH, but there was a significant effect of organic matter treatment (Table 2). We observed a linear increase in OC along the organic matter treatment gradient (r^2^ = 0.44, p < 0.001; Fig. 1b) with mean OC doubling from 2% in 0 g dw m^−2^ plots to 4% in 2250 g dw m^−2^ plots, and a linear decrease in porewater pH from 7.3 to 6.6 (r^2^ = 0.47, p < 0.001; Fig. 1c). Both OC and porewater pH exhibited high variability, particularly at the highest levels of organic matter treatment.

**Table 1:** Surface sediment (0–2 cm) characteristics in ambient, procedural control (PC) and treatment plots. Medians (and ranges) are given.

Treatment	Median grain size (µm)	Mud content (%)	Organic content (%)	Chlorophyll *a* (µg g^−1^ dw)	Phaeopigment (µg g^−1^ dw)
Ambient (n = 3)	189 (187–190)	2.6 (1.7–4.7)	1.7 (1.6–1.9)	10.3 (10.1–14.2)	5.1 (4.4–5.6)
PC (n = 4)	191 (185–195)	4.5 (2.3–5.7)	2.1 (1.9–2.2)	10.2 (8.2–14.5)	3.6 (3.4–5.7)
+CaCO_3_ (−OM) (n = 4)	193 (190–197)	3.1 (1.3–4.5)	1.8 (1.7–1.9)	11.5 (9.5–12.5)	3.6 (2.9–4.8)
+CaCO_3_ (+OM) (n = 14)	192 (177–199)	2.6 (1.1–6.1)	1.9 (1.7–3.0)	11.8 (8.9–20.3)	6.0 (4.3–22.7)
−CaCO_3_ (+OM) (n = 14)	191 (182–198)	2.8 (1.3–3.5)	2.0 (1.7–2.4)	11.6 (6.6–15.2)	5.7 (2.2–27.1)

**Table 2:** Results of PERMANOVA testing the direct and interactive effects of organic matter (OM, continuous covariable) and CaCO_3_ (fixed factor) treatments on sediment characteristics, macrofauna community and ecosystem functions.

		Source	Pseudo-F	p (perm)
Sediment characteristics				
OC (2–5 cm)		OM	26.1	**0.0001**
		CaCO_3_	0.61	0.45
		OM × CaCO_3_	0.26	0.62
Porewater pH		OM	28.9	**0.0001**
		CaCO_3_	0.03	0.87
		OM × CaCO_3_	0.003	0.96
Macrofauna				
Total abundance		OM	100	**0.0001**
		CaCO_3_	0.49	0.49
		OM × CaCO_3_	1.30	0.27
Total species		OM	38.6	**0.0001**
		CaCO_3_	1.15	0.29
		OM × CaCO_3_	1.98	0.17
Ecosystem functions				
NPP		OM	39.4	**0.0001**
		CaCO_3_	0.26	0.66
		OM × CaCO_3_	8.91	**0.007**
SOC		OM	0.52	0.48
		CaCO_3_	0.17	0.68
		OM × CaCO_3_	1.56	0.23
ln NH_4_^+^	Light	OM	62.8	**0.0001**
		CaCO_3_	0.001	0.98
		OM × CaCO_3_	1.69	0.21
	Dark	OM	0.61	0.44
		CaCO_3_	0.89	0.35
		OM × CaCO_3_	11.4	**0.002**
NO_x_	Light	OM	1.07	0.31
		CaCO_3_	3.12	*0.08*
		OM × CaCO_3_	0.04	0.84
	Dark	OM	0.06	0.80
		CaCO_3_	0.30	0.59
		OM × CaCO_3_	0.98	0.33

Bolded values indicate significant (p(perm) < 0.05) and italicised values indicate marginally significant (α < 0.1) effects. OC = organic content, NPP = net primary production, SOC = sediment oxygen consumption and ln NH_4_^+^  = natural log-transformed ammonium.

**Figure 2:**
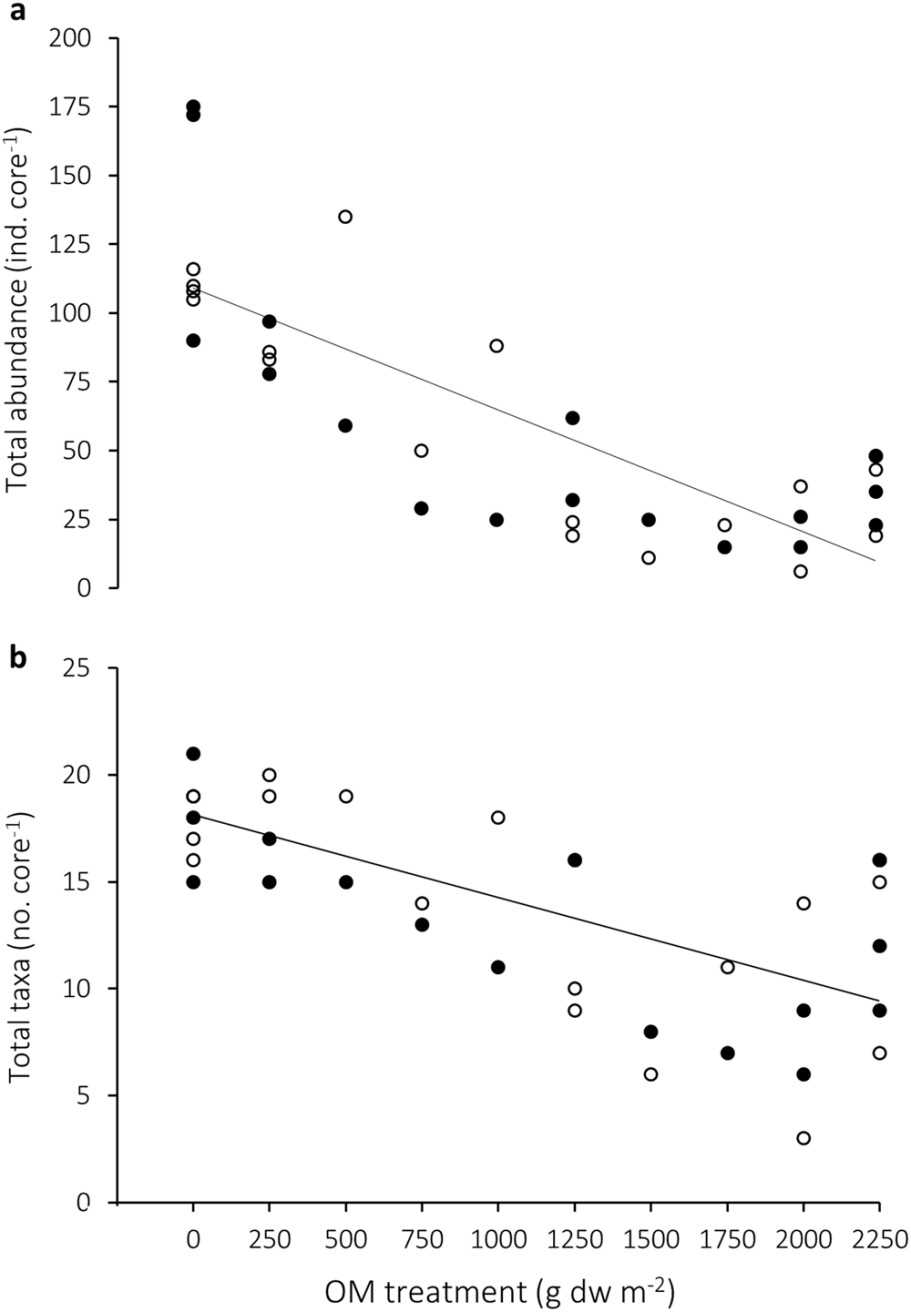
Macrofauna community response to organic matter (OM) treatment: (**a**) total abundance (r^2^ = 0.74, p < 0.001) and (**b**) total taxa (r^2^ = 0.52, p < 0.001). Closed symbols represent −CaCO_3_ treatments and open symbols represent +CaCO_3_ treatments, and regression lines are fitted to the pooled data set (see Table 2).

Macrofauna abundance data were averaged from two cores per plot, and total abundance and total number of taxa were determined per core (133 cm^2^). These community indices were only affected by organic matter treatment; CaCO_3_ treatment and its interaction with organic matter were both insignificant (Table 2). Mean total abundance decreased by 72% (r^2^ = 0.74, p < 0.001; Fig. 2a) and total species by 31% (r^2^ = 0.52, p < 0.001; Fig. 2b) along the organic matter treatment gradient.

**Figure 3:**
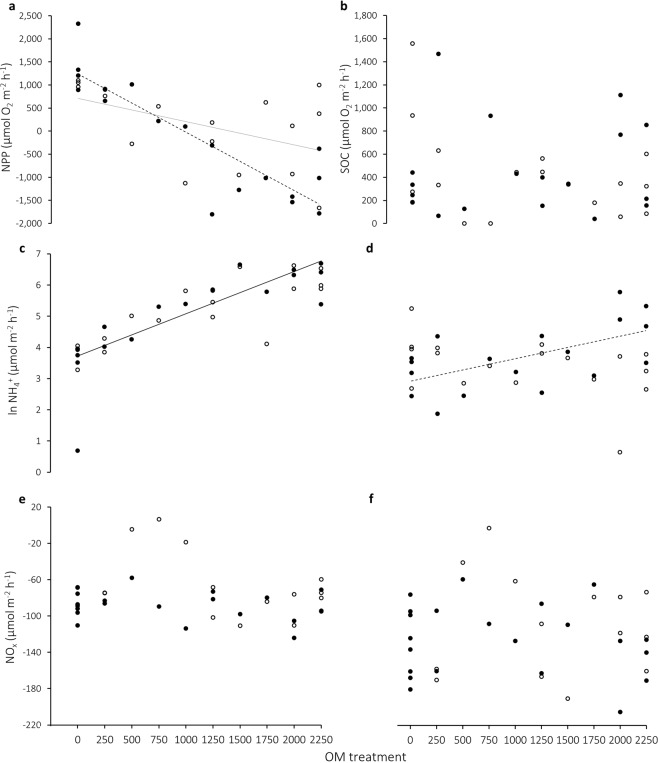
Response of ecosystem functions to organic matter (OM) and CaCO_3_ addition: (**a**) net primary productivity (NPP); (**b**) sediment oxygen consumption (SOC); (**c**) natural log-transformed ammonium (NH_4_^+^) efflux in the light and (**d**) dark; (**e**) combined nitrite and nitrate (NO_x_) uptake in the light and (**f**) dark. Closed symbols represent −CaCO_3_ treatments and open symbols represent +CaCO_3_ treatments. Significant (α < 0.05) linear regressions are indicated (see text for test statistics): dashed line = −CaCO_3_ treatment; solid grey = + CaCO_3_ treatment; solid black = combined CaCO_3_ treatments (i.e. no interaction between OM and CaCO_3_ treatments; see Table 2).

Fluxes of dissolved solutes (oxygen (DO), ammonium (NH_4_^+^), nitrite (NO_2_^−^) and nitrate (NO_3_^−^)) across the sediment–water interface were used to derive indicators of key ecosystem functions pertaining to primary productivity (PP), community metabolism and nutrient processing: light chamber DO flux was equivalent to net PP (NPP) and dark chamber DO flux corresponded to sediment oxygen consumption (SOC; a proxy for community metabolism). Fluxes of nitrogenous compounds indicated rates of nutrient cycling, with NH_4_^+^ being the first product of organic matter mineralisation which may be further oxidised into NO_2_^−^ and NO_3_^−^ (combined and represented as NO_x_). Treatment effects were not consistent across ecosystem functions; NPP and NH_4_^+^ flux exhibited significant relationships with organic matter treatment, whereas SOC and NO_x_ did not (Table 2). Chambers at low levels of enrichment were initially autotrophic (PP > SOC) during light incubations and became heterotrophic (SOC > PP) at higher levels of organic matter treatment (Fig. 3a). CaCO_3_ treatment significantly altered the response of NPP to organic matter enrichment (Table 2), with +CaCO_3_ plots exhibiting net autotrophy to higher levels of organic matter treatment than −CaCO_3_ plots (~1400 versus ~950 g dw m^−2^). NPP decreased significantly with increasing organic matter treatment in −CaCO_3_ plots (r^2^ = 0.77, p < 0.001), whereas the loss in productivity in +CaCO_3_ plots was less steep, more variable and was only marginally correlated with organic matter treatment (r^2^ = 0.24, p = 0.05). SOC showed no relationship with organic matter or CaCO_3_ treatments (Fig. 3b, Table 2) with high variability across the range of organic matter treatments.

Under light conditions natural log-transformed NH_4_^+^ flux (NH_4_^+^_light_) showed a significant response to organic matter treatment, but not CaCO_3_ or its interaction with organic matter treatment (Table 2). As organic matter enrichment increased, the rate of NH_4_^+^_light_ efflux from sediments increased exponentially (r^2^ = 0.65, p = < 0.001; Fig. 3c), such that the rate of NH_4_^+^_light_ efflux was more than 500 times greater in 2250 versus 0 g dw m^−2^ treatments. In light chambers, NO_x_ (NO_x light_) showed a marginally significant response to CaCO_3_ addition (but not organic matter treatment; Table 2), with a slight decrease in net uptake (~15 µmol m^−2^ h^−1^) in +CaCO_3_ treatments (Fig. 3e). Under dark conditions (i.e. in the absence of microphytobenthos (MPB) uptake), there was a significant relationship between organic matter treatment and natural log-transformed NH_4_^+^ (NH_4_^+^_dark_) efflux which changed with CaCO_3_ treatment, as indicated by the significant interaction term (Table 2). In −CaCO_3_ treatments, efflux of NH_4_^+^_dark_ increased exponentially as organic matter treatment increased (r^2^ = 0.35, p = 0.01; Fig. 3d), however this relationship was absent in +CaCO_3_ treatments and efflux rates were generally reduced. There was far greater variability in nutrient cycling measurements obtained under dark versus light conditions.

When constructing multiple regression models, marginal tests first identified significant (p(perm) <0.1) predictors of response variables when considered alone, and full models then determined the best combination of predictor variables after accounting for the variance attributed to organic matter treatment (organic matter treatment was always fitted first). In marginal tests, variability in ecosystem functions was correlated with several variables including organic matter treatment, sediment characteristics and macrofauna community indices (Table 3). For functions that exhibited a significant interaction between organic matter and CaCO_3_ treatments (NPP and NH_4_^+^_dark_), the −CaCO_3_ treatment had a greater number of significant predictors that individually explained more of the variation than the +CaCO_3_ treatment. When organic matter treatment was fitted first, it was the only predictor included in backwards elimination linear regression models of NPP for both CaCO_3_ treatments, but the amount of variance explained decreased from 76% in −CaCO_3_ treatments to 21% in +CaCO_3_ treatments. Although macrofauna total abundance and porewater pH explained large amounts of variance in NPP (−CaCO_3_ treatments) in marginal tests, both variables were highly correlated with organic matter treatment (r > 0.8) and were excluded from the model. The variables included in the full model of NH_4_^+^_dark_ flux differed depending on CaCO_3_ treatment. A combination of organic matter treatment and sediment characteristics (CaCO_3_ content and phaeopigments) together explained 68% of the variance in −CaCO_3_ treatments, whereas only 17% of the variance was explained in the full model for +CaCO_3_ treatments by organic matter treatment. In marginal tests total species explained more of the variation in +CaCO_3_ treatments than organic matter treatment, but was excluded from the full model. For NH_4_^+^_light_ flux, despite numerous variables being identified as significant predictors when considered alone, only two were included in the full model; organic matter treatment contributed most of the total variance explained (66 of 71%), with the remainder attributed to chlorophyll *a* (Chl *a*), an indicator of MPB abundance.

**Table 3:** Results of DistLM determining best combinations of predictor variables (backwards elimination procedure) explaining ecosystem function response to organic matter (OM) treatment.

Function	Treatment	Variable	Pseudo-F	Proportion	Total
NPP	−CaCO_3_	**OM treatment**	**60.3**	0.76***	
		Total abundance	20.8	0.52***	
		Porewater pH	18.0	0.49***	
		Phaeopigments	8.63	0.31***	
		OC 2–5 cm	7.38	0.28***	
		Total species	7.46	0.28**	
		Mud content	4.74	0.2**	
					**76%**
	+CaCO_3_	**OM treatment**	**7.01**	0.21***	
		Total abundance	10.6	0.4***	
		Porewater pH	3.64	0.19*	
					**21%**
Ln Dark	−CaCO_3_	**OM treatment**	**9.76**	0.34***	
NH_4_^+^ flux		**Phaeopigments**	**10.1**	0.35***	
		**CaCO**_**3**_ **content**	**5.86**	0.24**	
		Total abundance	4.93	0.21**	
		Total species	3.39	0.15*	
					**68%**
	+CaCO_3_	**OM treatment**	**3.34**	0.17 *	
		Total species	6.61	0.29**	
		Total abundance	4.1	0.2*	
					**17%**
Ln Light		**OM treatment**	**73.1**	0.66***	
NH_4_^+^ flux		Total abundance	51.7	0.58***	
		Porewater pH	18.4	0.33***	
		OC 2–5 cm	15.6	0.3***	
		Total species	14.4	0.28***	
		Phaeopigments	5.19	0.12**	
		**Chl** ***a***	4.89	0.12**	
					**71%**

OM treatment was fitted first in all models and CaCO_3_ treatments are analysed separately where significant interactions between OM and CaCO_3_ treatments occur. Proportion gives the amount of variability explained by each variable when considered alone (marginal tests). Variables in bold were included in the best full models and total shows the total variation explained by full models. Level of significance: *p < 0.1, **p < 0.05, ***p < 0.01.

## Discussion

Natural fluctuations in pH may be extreme in the benthos of productive estuarine environments^8^, however coastal eutrophication and global OA are set to intensify acidification across varying scales of magnitude, space and time such that benthic ecosystem functions may be compromised. For the first time an *in situ* manipulation of porewater pH and investigation of the ability of CaCO_3_ to prevent negative change has shown that CaCO_3_ deposits may increase the functional resilience of benthic ecosystems to localised eutrophication-induced acidification.

The decline of benthic biodiversity with increasing inputs of organic matter is well-documented and usually attributed to the inducement of hypoxic conditions^17,52^, however the potential for co-occurring porewater acidification to drive these declines has been comparatively under-studied. The highest organic matter treatment resulted in a porewater pH of 6.6, and the loss of one third of macrofauna species and almost three quarters of total individuals. No increase in porewater pH could be detected in +CaCO_3_ treatments, nor any difference in the relationship between macrofauna community indices and organic matter treatment. This suggests that CaCO_3_ did not buffer porewater pH sufficiently to decrease the physiological stress of the organisms, or that other consequences of organic matter degradation such as hypoxia or increased [H_2_S] were more important in driving the macrofaunal response. The latter seems most likely, given CaCO_3_ dissolution under open-system conditions would serve to maintain pH within a given range, rather than drive it higher than typical pore water values^43^.

These biodiversity losses were associated with a predictable decrease in function, given biodiversity–ecosystem functioning relationships are frequently density-dependent^53^ and/or underpinned by key species^54,55^. NPP decreased significantly along the organic matter treatment gradient whilst NH_4_^+^_light_ efflux increased, indicating a decrease in the utilisation of nutrients by the MPB community. Chl *a* biomass, a proxy for MPB abundance, showed no relationship with organic matter treatment and therefore cannot explain trends in NPP. It has been hypothesised that increased pCO_2(aq)_ would result in increased productivity of photoautotrophs, and although most research has focussed on seagrasses and macroalgae^36,56,57^, increases in the PP of MPB have been observed in the laboratory following exposure to CO_2_-acidified seawater^37,57^. The unfavourable sediment conditions (low pH, low [O_2_]) created here may have stressed the MPB community beyond being able to take advantage of excess CO_2_. The decrease in abundance of macrofauna, whose activities are known to stimulate MPB PP^58^, may also have played a role. However, CaCO_3_ addition increased the resilience of NPP to these conditions. Significant increases in pH, total alkalinity (TA) and saturation states have been measured previously as a result of biogenic CaCO_3_ dissolution^44,47,51^, so the coarse methods used to measure pH changes here (pH field probe with ±0.2 pH units accuracy) may have masked finer scale alterations to porewater biogeochemistry which benefitted the MPB community. Importantly, CaCO_3_ addition had the effect of maintaining autotrophy of the benthic ecosystem to a considerably higher level of organic matter treatment, and this effect was strong enough to be detected amid the complexity of the field.

The response of light and dark nutrient cycling to organic matter treatment (increasing efflux of NH_4_^+^ and no response of NO_x_) indicates a limitation or breakdown of microbially-mediated nitrification pathways that oxidise NH_4_^+^ to NO_2_^−^ and NO_3_^−^. There are several ways that organic matter treatment may have limited nitrification, for example a decrease in bioturbation and bioirrigation activity driven by the decrease in macrofaunal abundance may have reduced the availability of aerobic sediments^59^, which are required by microbial nitrifiers (i.e. ammonia- and nitrite-oxidisers). However, Braeckman *et al*.^60^ have previously shown that in the short-term (14 d), decreases in benthic nitrification rates under acidified conditions are mediated predominantly by changes in microbial community activity, rather than macrofaunal facilitation. Inhibition of nitrifiers at low pH may therefore be a factor, although it is expected that microbes are tolerant to considerable fluctuations in pH and Kitidis *et al*.^61^ previously found no significant decrease in nitrification rates at pH 6.1. Substrate-limitation is perhaps the most likely cause as NH_3_ is the true substrate for ammonia-oxidisers, yet below pH ~8.0 NH_3_ is present almost exclusively as NH_4_^+^ ^62,63^. Alternatively, biogeochemical processes such as dissimilatory NO_x_ reduction to NH_4_^+^ may have increased [NH_4_^+^], a process which can be quantitatively more important than denitrification in organically-rich sediments^64,65^.

CaCO_3_ addition altered the relationship between nutrient cycling and organic matter treatment (in the dark only), such that there was no trend of increasing NH_4_^+^ efflux in +CaCO_3_ treatments. As porewater O_2_ would have been scarce in dark conditions (absence of photosynthesis) and CaCO_3_ addition had no effect on the macrofaunal community, it is unlikely that the improvement to sediment conditions that helped maintain nitrification rates was due to increasing availability of aerobic sediments. Similarly, there was no detected increase in pH with CaCO_3_ addition, therefore NH_3_ availability should be equally limiting. It seems most likely, therefore, that subtle changes to porewater biogeochemistry that were not measured played a role in improving conditions for nutrient cycling microbes. In future research it will be important to monitor a larger suite of carbonate chemistry parameters (pH, TA, dissolved inorganic carbon (DIC)) to identify the mechanisms underlying these functional responses. However, the aim of this study was to investigate whether laboratory-based theory could be applied in the field, and indeed, CaCO_3_ addition successfully dampened the negative effects of organic matter enrichment on nutrient cycling. This justifies further investigation of these interactions *in situ* and has important consequences for the functioning of coastal ecosystems, given the conversion of biologically-available nitrogen to nitrogenous gas provides resilience against further eutrophication^66,67^.

Whilst some ecosystem functions responded strongly to organic matter treatment, SOC showed no relationship with enrichment, OC or porewater pH. Since SOC is the net result of biological (BOD) and chemical oxygen demand (COD)^68^, it was hypothesised that SOC would increase with organic matter treatment due to an increased BOD of the stimulated microbial community and COD from increasing abundance of reducing substances, as has previously been reported for organically enriched sediments (e.g.^69–71^). The lack of a relationship may be explained by a ‘compensation’ in O_2_ consumption along the organic matter treatment gradient, whereby the gradual decrease in respiration and O_2_ consumption facilitated by macrofauna (due to decreasing abundance and stress-induced change in activity levels) was offset by a gradual increase in microbial respiration and COD, such that overall SOC remained constant.

In −CaCO_3_ treatments combinations of organic matter treatment, sediment characteristics and macrofauna community variables were important predictors of NPP and NH_4_^+^_dark_ flux. In +CaCO_3_ treatments far fewer variables were significant predictors of function, though macrofauna community indices remained significant and individually explained more variation than organic matter treatment. This implies a strong facilitation of ecosystem function by the macrofauna community even when the geochemical complexities of sediments are enhanced. Organic matter treatment was the only predictor included in several full models (when fitted first), highlighting the dominant effect of this stressor on ecosystem function, however there was a substantial decrease in the variance it explained in +CaCO_3_ treatments compared to −CaCO_3_. This discrepancy may have been associated with the microbial component of the ecosystem, as it is the microbial community that converts and transforms solutes, though the macrofauna and sediment characteristics are key controllers of solute transport rates and microbial microhabitats^72^. Changes to porewater chemistry may also have driven the responses observed, therefore including a greater number of carbonate chemistry parameters and microbial community indicators in models may result in greater explanation of the functional response.

In this short-term, single-application experiment, CaCO_3_ effectively reduced the negative effects of organic matter enrichment on ecosystem function. However, over longer timeframes and repeated CaCO_3_ applications there may be potential effects associated with its addition. The extent of these effects will likely be dependent upon the difference in grain size between the ambient sediment and the added CaCO_3_, and whether this difference substantially alters grain size distribution. If so, benthic ecosystem functions could be affected as diffusive solute exchange across the sediment–seawater interface is highly dependent upon sediment permeability^73^, and the effects of macrofaunal bioturbation on function change along gradients of sediment grain size^74^. To avoid such impacts CaCO_3_ should be crushed or ground to match the sediment characteristics of the receiving site prior to addition, whilst keeping in mind that the dissolution rates of CaCO_3_ will vary as a function of grain size^44^.

## Conclusions

Benthic ecosystem functions respond demonstrably to experimentally elevated organic matter and CaCO_3_ content in a field setting, despite complex interactions occurring between levels of biological organisation and environmental characteristics that influence function. Porewater acidification resulting from organic matter enrichment has negative effects on primary production and nutrient cycling which are dampened by the addition of biogenic CaCO_3_, thus increasing the resilience of the ecosystem to this stressor. The lack of a beneficial effect of CaCO_3_ on macrofauna biodiversity suggests other stressors associated with eutrophication (hypoxia, H_2_S toxicity) were stronger determinants of community structure than pH. The positive effects of CaCO_3_ addition may therefore be microbially mediated, and future research should focus on the combined response of macro- and microbiological communities to coastal eutrophication *in situ*. Developing a mechanistic understanding of how CaCO_3_ provides resilience to ecosystem function will be crucial, as losses of key CaCO_3_-producing species are likely under future coastal acidification stressors.

## Methods

The experiment was conducted in the mid-intertidal zone of Tuapiro estuary (37°29.406′, 175°57.074′) within Tauranga Harbour, New Zealand. The site experiences a diurnal tidal cycle with inundation periods of ~5 h and a mean water depth ~1.2 m upon maximum inundation. Plots were established within a 12 m × 30 m (360 m^2^) area of homogeneous fine sand (median grain size 191 µm) with low mud (silt/clay particles <63 µm) (<5%), CaCO_3_ (<1%) and organic content (1.6–2.2% in un-enriched plots) (Table 1).

In austral summer (31^st^ January 2017), forty 1.44 m^2^ plots were established (2 m apart) in a 10 column by 4 row array. Four replicates of each of the following three treatments were randomly allocated: ambient (no treatment), procedural control (PC; sediment disturbed mimicking treatment setup) and plots that received CaCO_3_ (pure crushed oyster shell, fragments <5 mm) but no organic matter (+CaCO_3_ (−OM)). The remaining 28 plots were randomly assigned one of nine organic matter enrichment treatments created using 250–2250 g dw m^−2^ of organic plant-derived slow-release garden fertiliser (treatments increasing in 250 g increments in a gradient design). Enriched plots were also assigned a CaCO_3_ treatment (with or without the addition of 2000 g dw m^−2^). Replication was carried out for the lowest (250 g m^−2^ (n = 3)), middle (1250 g m^−2^ (n = 2)) and highest (2000 (n = 2) and 2250 g m^−2^ (n = 3)) organic matter treatments. The materials used to create organic matter and CaCO_3_ treatments were chosen to mimic natural inputs to estuarine systems, whilst slow-release fertiliser ensured sustained organic matter degradation would result in alterations to the biogeochemical properties of the sediment.

Plots were excavated to 5 cm depth, taking care to minimise disruption to the sediment matrix, before evenly distributing organic matter and CaCO_3_ (in the +CaCO_3_ treatments) across plots and replacing sediment. The organic matter enrichment range was chosen to create a gradient in sediment OC that would elicit a response typical of eutrophically-stressed systems (i.e. acidified porewaters and reduced biodiversity), and represents a one-off depositional event such as a macroalgal bloom collapse. Such events are common globally (e.g.^75,76^) and in the wider estuary due to seasonal *Ulva* spp. blooms^77^. The level of CaCO_3_ enrichment elevated the average ambient content at the study site by 10x, which is representative of the use of CaCO_3_ as an acidification mitigation tool rather than a natural increase in CaCO_3_ content. Comparisons of sediment characteristics, macrofaunal community indices and benthic solute fluxes between ambient, procedural and CaCO_3_ control plots confirmed there was no significant effect associated with the presence of the added CaCO_3_ or the procedure of adding it to the sediment (see Supplementary Information).

Benthic solute fluxes and sediment properties were measured *in situ* after nine weeks (3^rd^–4^th^ April 2017, early austral autumn), and macrofauna communities were sampled the following week (11^th^ April 2017), allowing time for changes in the sediment biogeochemistry and macrofauna community to occur. Benthic flux incubations were conducted over two days with midday high tides. Fluxes of dissolved solutes across the sediment–water interface were measured under light and dark conditions in each plot, i.e. in the presence and absence of photosynthesis. As rates of photosynthesis vary with light intensity^78^, all light measurements were conducted on day one (and dark measurements on day two) so that chambers experienced the same light regime^79^. One ambient plot was dropped from the analysis due to a chamber malfunction.

The operation of the incubation chambers used for measuring benthic fluxes has previously been described^80–82^. Briefly, plots were divided into quarters and chamber bases (L50 × W50 × H15 cm) were positioned in a randomly chosen quarter ~10 cm from plot edges, and inserted 5 cm into the sediment. On day 2 (dark incubations), chambers were placed diagonally opposite to the day 1 position to minimise disturbance effects. HOBO Pendant temperature and light loggers (Onset) were installed in 16 of the chambers dispersed across the site, with an additional three secured on the seafloor (10 s sampling frequency). Domed Perspex lids were clamped onto bases on the incoming tide, encapsulating ~34 L of water. Lids were transparent for light incubations, whilst double-layered shade cloth covered lids for dark incubations. Previous measurements using HOBO loggers have recorded zero light inside chambers covered using this method (V. Rullens, unpublished data). Syringe-drawn water samples (60 mL) were collected in triplicate at the start and end of the ~4-h incubation. Ambient seawater samples (n = 3) were collected as chambers were sealed, and 3 light and dark pairs of 1 L bottles were filled and secured at the seafloor for the duration of the incubations; sampling the bottles at the end of incubations allowed determination of the contribution of water column processes to solute fluxes. Water column processes accounted for <7% of benthic solute fluxes in both light and dark conditions and were therefore ignored. Seawater DO was measured immediately using an optical probe (PreSens FIBOX 3 LCD trace v7); a two-point calibration was performed prior to measurements in a Na_2_SO_4_ O_2_-free (0%) solution and air-saturated water (100%) following the manufacturer’s instructions. Samples were then filtered through 0.8 µm glass fibre filters (Whatman GF/F) and stored frozen, in the dark prior to analysis of dissolved nutrient concentrations.

Weather conditions varied substantially between days of benthic flux measurements; day 1 was sunny with little wind wave activity whereas day 2 was cloudy with intermittent rain and some wind wave activity. Although the cloudier conditions experienced on day 2 were irrelevant in terms of incident sunlight (day of dark incubations), mean water temperature inside chambers differed between day 1 (23 °C) and 2 (19 °C), which could have impacted solute fluxes.

Sediment cores were collected from within each plot to characterise sediment properties. Six small cores (2.6 cm diameter × 2 cm depth) were pooled per plot and stored frozen, in the dark for analysis of sediment grain size and indicators of MPB biomass (chlorophyll *a* (Chl *a*) and phaeopigments (phaeo)). Four medium cores (5 cm diameter × 7 cm depth) were collected and 0–2 cm and 2–5 cm sections were pooled for OC analysis at both depth intervals and CaCO_3_ content at 2–5 cm (depth of burial). Two large cores (13 cm diameter × 15 cm depth) for macrofaunal analysis were collected and sieved *in situ* over a 500 µm mesh, preserved in 70% isopropyl alcohol. Finally, porewater pH was measured in each plot using a digital field probe (Eutech pHTestr1, 2; accurate to ±0.2 pH units with a tip diameter of 3 mm) at ~2 cm depth following calibration using pH 4 and 7 standard solutions.

Sediment samples were thawed, homogenised and divided for analysis of sediment properties. Grain size samples (~10 g) were digested in 10% hydrogen peroxide to remove organic matter before measuring using a Malvern Mastersizer 2000 (particle size range 0.05–2000 µm). Sediment pigment samples (Chl *a* and phaeo) were processed using standard procedures^83^ and analysed on a Turner 10–AU fluorometer using an acidification step. Percent organic and CaCO_3_ contents were determined from 5 g dried sediment samples (100 °C until constant weight) according to standard loss on ignition (LOI) procedures for OC^84^ and CaCO_3_^85^. Macrofauna samples were stained with Rose of Bengal and identified to lowest possible taxonomic level (usually species). Filtered water samples were thawed and analysed for dissolved inorganic nutrient species (ammonium (NH_4_^+^), nitrite (NO_2_^−^) and nitrate (NO_3_^−^)) using standard colorimetric and fluorometric techniques (Astoria 2 Pacific autoanalyser).

Dissolved oxygen and nutrient fluxes (µmol m^−2^ h^−1^) were calculated by subtracting the final concentration (µmol L^−1^) of the given solute from the initial concentration and standardising by the incubation duration (h), chamber water volume (L) and enclosed sediment surface area (m^−2^). These fluxes underpinned the quantification of key ecosystem functions: light chamber DO flux was equivalent to net PP (NPP) and dark chamber DO flux was equivalent to sediment oxygen consumption (SOC; a proxy for community metabolism), while fluxes of nitrogenous compounds indicated rates of nutrient cycling. Concentrations of NO_2_^−^ and NO_3_^−^ were frequently below detection limits and are thus combined and represented as NO_x_.

In all analyses, organic matter treatment (i.e. the quantity of organic matter initially added to plots) was used as the continuous independent variable against which responses were measured. Using organic matter treatment better represented the initial stress experienced by the benthic ecosystem, whereas OC (measured at the time of flux incubations) alternatively indicated the degree of organic matter degradation that had occurred since plot establishment, which may vary according to the natural heterogeneity of the environment.

To determine whether organic matter and CaCO_3_ treatments (incorporated at 5 cm sediment depth) had altered sediment characteristics as intended, OC (2–5 cm) and porewater pH were regressed against organic matter treatment, and a *t*-test was performed to detect differences in CaCO_3_ content (2–5 cm) between +CaCO_3_ and −CaCO_3_ plots. Simple linear regression identified significant (α < 0.05) responses of the macrofauna community and ecosystem functions to organic matter treatment (using Statistica v13).

To simultaneously account for the gradient in organic matter treatment and assess the effects of CaCO_3_ addition on sediment characteristics, macrofauna community and ecosystem functions, PERMANOVA (using PERMANOVA+, Primer V7) were performed based on Euclidean distances with CaCO_3_ treatment considered a fixed factor and organic matter treatment a continuous co-variable. Where a significant interaction (p(perm) <0.05) occurred −CaCO_3_ and +CaCO_3_ treatments were considered separately for subsequent analyses, in the absence of a significant interaction the treatments were pooled. As PERMANOVA obtains p-values through permutation and does not assume normality, response variables were untransformed. For constructing DistLM, predictor variables were normalised and highly correlated variables (Pearson’s r > 0.8) were identified; the variable explaining the lesser proportion of variance was excluded from model selection to avoid collinearity^86^. Full models used a backward elimination procedure with the corrected Akaike’s Information Criterion (AICc) and 9999 permutations to obtain the most parsimonious model^87^.

## Supplementary information

Calcium carbonate alters the functional response of coastal sediments to eutrophication-induced acidification

## Data Availability

Upon acceptance of this manuscript, the data supporting the results will be archived in an appropriate public repository and the data DOI will be included at the end of the article.
