# Supplementary material for: Calcium carbonate alters the functional response of coastal sediments to eutrophication-induced acidification
Source: Sci Rep. 2019 Aug 19;9:12012. doi: 10.1038/s41598-019-48549-8 (PMC6700140; doi:10.1038/s41598-019-48549-8)
Supplement: Supplementary file 1 — Calcium carbonate alters the functional response of coastal sediments to eutrophication-induced acidification [file 41598_2019_48549_MOESM1_ESM.pdf]

Supplementary information

Calcium carbonate alters the functional response of coastal sediments to eutrophication-induced acidification

Tarn P. Drylie, Hazel R. Needham, Andrew M. Lohrer, Adam Hartland, Conrad A. Pilditch

Our study utilised three different control treatments to account for potential effects of experimental procedures; a procedural control to mimic the disturbance associated with treatment setup, a $CaCO_3$ control (+$CaCO_3$ –OM) to identify effects of the physical presence of $CaCO_3$, and un-manipulated ambient plots that acted as reference for comparison.

Seventy days after establishment $CaCO_3$ control plots had a higher $CaCO_3$ content than the ambient and procedural control plots, as expected due to the addition of $CaCO_3$, and phaeopigment concentrations were higher in ambient than procedural and $CaCO_3$ controls (Table S1). When tested using one-way PERMANOVA, no surface sediment characteristics (median grain size, organic and mud content, chlorophyll a, phaeopigments and porewater pH) differed significantly (p(perm) > 0.05) between control treatments (results not shown), nor did a multivariate measure combining these variables (Table S2). There was a marginally significant difference in the abundance of adult *Austrovenus stutchburyi* between control treatments (Table S2) driven by a lower abundance in PC plots, however there was no such effect on *Macomona liliana*; total abundance and the number of taxa were also comparable between treatments (Table S1).

There was no consistent variation in measures of ecosystem function and nitrogenous solute fluxes between control treatments (Table S1). Although net primary production (NPP) was greatest in PC treatments, and sediment O_2 consumption (SOC) was highest in $CaCO_3$ controls, high varibility (consistent with the dynamic nature of the sandflat) meant there was no significant differences between treatments (NPP: Pseudo-F = 2.80, p(perm) = 0.12; SOC: Pseudo-F = 1.58, p(perm) = 0.22). There was little difference in the average flux of nitrogenous solutes between control plots (Table S1) and a single multivariate measure of ecosystem function (consisting of NPP, SOC and nitrogenous solute fluxes) did not differ between treatments (Table S2).

Overall, no consistent differences were detected between our control treatments with respect to sediment characteristics, macrofaunal community composition or ecosystem function. Therefore, apart from raising sediment $CaCO_3$ content, the procedure of removing and replacing sediment and incorporating $CaCO_3$ did not substantially affect the response variables measured in this study.

Table S1. Mean (and standard deviation) of surface sediment characteristics (0–2 cm unless stated otherwise), macrofaunal community indices and solute fluxes in ambient, procedural control (PC) and $CaCO_3$ control plots. Abbreviations and units: Organic content (OC), $CaCO_3$ content and mud content, %; Chlorophyll *a* and phaeopigments, µg g^{-1} dw sediment; median grain size, µm; macrofauna community indices, no $core^{-1}$; all ecosystem functions, µmol m^{-2} h^{-1}.

	Ambient (n = 3)		PC (n = 4)		$CaCO_3$ (n = 4)	
Sediment characteristics						
OC	1.7	(0.1)	2.1	(0.1)	1.9	(0.1)
OC (2–5 cm)	2.0	(0.2)	2.0	(0.4)	2.2	(0.1)
$CaCO_3$ (2–5 cm)	0.5	(0.1)	0.7	(0.4)	1.1	(0.4)
Mud content	3.0	(1.5)	4.2	(1.4)	3.1	(1.1)
Chlorophyll *a*	11.5	(2.3)	10.8	(2.6)	11.3	(1.3)
Phaeopigments	5.0	(0.6)	4.1	(1.1)	3.3	(0.5)
Median grain size	188	(2)	190	(4)	192	(2)
Porewater pH (at 2 cm depth)	7.4	(0.2)	7.3	(0.3)	7.3	(0.2)
Macrofauna community						
Total abundance	133	(31)	109	(25)	113	(33)
Total taxa	23	(1)	23	(3)	22	(1)
A. stutchburyi (>5 mm)	9	(2)	5	(2)	8	(2)
M. liliana (>5 mm)	4	(1)	3	(1)	4	(1)
Ecosystem functions						
NPP	682	(34)	1440	(621)	988	(102)
SOC	471	(145)	302	(111)	776	(601)
$NH_4^+{}_{light}$	17	(17)	6	(22)	-8	(14)
$NH_4^+{}_{dark}$	19	(15)	17	(13)	31	(24)
$NO_2^-{}_{light}$	5	(5)	7	(3)	2	(3)
$NO_2^-{}_{dark}$	2	(4)	-6	(4)	-2	(3)
$NO_3^-{}_{light}$	-108	(23)	-89	(14)	-88	(17)
$NO_3^-{}_{dark}$	-139	(36)	-116	(48)	-131	(31)

Table S2. Results of one-way PERMANOVAs comparing multivariate sediment characteristics and ecosystem function, and univariate macrofaunal community indices between control (ambient, procedural and $CaCO_3$) treatments.

	df	Pseudo-F	p(perm)
Sediment characteristics*	2	0.92	0.45
Macrofauna community			
Total abundance	2	0.48	0.65
Total taxa	2	0.43	0.69
A. stutchburyi >5 mm	2	4.51	0.05
M. liliana >5 mm	2	0.91	0.39
Ecosystem function*	2	2.11	0.08

*Sediment characteristics and ecosystem function encompass all corresponding variables included in Table S1.
